# The Role of β-Carotene in Colonic Inflammation and Intestinal Barrier Integrity

**DOI:** 10.3389/fnut.2021.723480

**Published:** 2021-09-27

**Authors:** Junrui Cheng, Emilio Balbuena, Baxter Miller, Abdulkerim Eroglu

**Affiliations:** ^1^Plants for Human Health Institute, North Carolina State University, Kannapolis, NC, United States; ^2^Department of Molecular and Structural Biochemistry, College of Agriculture and Life Sciences, North Carolina State University, Raleigh, NC, United States

**Keywords:** β-carotene, colonic inflammation, colonic epithelial cells, tight junctions, vitamin A

## Abstract

**Background:** Carotenoids are naturally occurring pigments accounting for the brilliant colors of fruits and vegetables. They may display antioxidant and anti-inflammatory properties in humans besides being precursors to vitamin A. There is a gap of knowledge in examining their role within colonic epithelial cells. We proposed to address this research gap by examining the effects of a major dietary carotenoid, β-carotene, in the *in vitro* epithelial cell model.

**Methods:** We examined the function of β-carotene in the lipopolysaccharide (LPS)/toll-like receptor 4 (TLR4) signaling pathway. We conducted western blotting assays to evaluate expressions of TLR4 and its co-receptor, CD14. We also examined NF-κB p65 subunit protein levels in the model system. Furthermore, we studied the impact of β-carotene on the tight junction proteins, claudin-1, and occludin. We further carried out immunocytochemistry experiments to detect and visualize claudin-1 expression.

**Results:** β-Carotene reduced LPS-induced intestinal inflammation in colonic epithelial cells. β-Carotene also promoted the levels of tight junction proteins, which might lead to enhanced barrier function.

**Conclusions:** β-Carotene could play a role in modulating the LPS-induced TLR4 signaling pathway and in enhancing tight junction proteins. The findings will shed light on the role of β-carotene in colonic inflammation and also potentially in metabolic disorders since higher levels of LPS might induce features of metabolic diseases.

## Introduction

Carotenoids are naturally occurring pigments responsible for the brilliant colors of fruits, vegetables, and microalgae (1, 2). Along with the carotenoids containing 30, 45, or 50 carbon atoms that are mainly observed in archaea and bacteria via biosynthesis (3, 4), the most abundant carotenoids in nature are C40 carotenoids (4, 5), which are formed with the cyclization, isomerization, and oxidation of eight C5 isoprenoid units (4, 6, 7). As one of the C40 carotenoids, β-carotene is the most prevalent carotenoid in North American diets and detected in human serum (8, 9); thus it is widely studied, especially for its potential beneficial effects in humans. Chemically, β-carotene is a hydrocarbon carotenoid, composed of the long chain of alternating double bonds (polyene chain) and β-ionone rings at both terminal groups (10). From the biological point of view, β-carotene is one of the major dietary provitamin A carotenoids, meaning that it can be cleaved by beta-carotene-15,15′-oxygenase 1 (BCO1) at the central double bond of the polyene chain to yield two molecules of all-trans retinal, which can be further oxidized to all-trans retinoic acid (ATRA), the main biologically active form of vitamin A, or reduced to all-trans retinol (11). In addition to central (symmetrical) cleavage, eccentric cleavage can take place asymmetrically resulting in the formation of β-apo-10'-carotenal and β-ionone (10). Other apocarotenoids, such as β-apo-8'-carotenal, β-apo-12'-carotenal, and β-apo-14'-carotenal, can also be formed enzymatically and non-enzymatically (10). Physiologically within humans, β-carotene is released from the food matrix and incorporated into mixed micelles in order to be absorbed within intestinal cells. Moreover, this cellular uptake occurs through a mechanism of passive diffusion or facilitated transport via scavenger receptor class B type 1 (SR-B1) (12). Finally, β-carotene is incorporated into chylomicrons for secretion and transport into the lymph (13).

Besides being a precursor to vitamin A, β-carotene can exert antioxidant and anti-inflammatory properties in humans. The anti-inflammatory effects of β-carotene have been demonstrated in multiple systems. Schultz et al. showed a reverse correlation between plasma β-carotene and C-reactive protein (CRP), a systemic inflammatory biomarker (14). Additionally, by administrating β-carotene-^14^C intraduodenally, Crain et al. reported that appreciable amounts of β-carotene was converted to ATRA in the rat intestinal mucosa (15), highlighting the possibility that both β-carotene and ATRA could be bioactive compounds that affected the homeostasis of the gastrointestinal (GI) system. Another study showed that subjects containing lower levels of β-carotene had increased levels of CRP and interleukin-6 (IL-6) (16), an inflammatory cytokine that contributes to the pathogenesis of various GI diseases (17, 18). Furthermore, multiple mechanistic studies were conducted to explore the signaling pathways involved in the anti-inflammatory activity of β-carotene. Among those, β-carotene was noted for inhibition of NF-κB translocation by blocking the activation of the NF-κB p65 subunit (19), leading to decreased transcription of pro-inflammatory cytokine genes such as interelukin-1β (IL-1β), IL-6, and tumor necrosis factor alpha (TNF-α) (20). Regarding a causative aspect of inflammation, lipopolysaccharide (LPS) is a surface molecule derived from the outer membrane of almost all gram-negative bacteria (21). Accumulating evidence has revealed that LPS can induce pathogen recognition receptors (PRRs), such as TLR4, which subsequently activates NF-κB, leading to the translocation of NF-κB from the cytoplasm to the nucleus (22). The mammalian NF-κB has various subunits including p65, p50, c-Rel, RelB that may form different types of hetero- or homodimers (23), but the p50/p65 heterodimer is the most common (24). NF-κB contributes to various pro-inflammatory activities due to its ability to transcriptionally activating pro-inflammatory cytokines including IL-6, IL-1β, and TNF-α (25).

Tight junctions are multiprotein cell-cell adhesion complexes whose functions are regulated by various tight junction proteins, which together help maintain the integrity of the intestinal barrier (26). Tight junction proteins including claudin-1, claudin-3, and claudin-15 significantly decreased, leading to increased intestinal permeability in a high-fat diet-induced obese mice model (27). A leaky gut allows the release of detrimental exterior molecules from the GI tract to the host, facilitating the progression of systemic inflammation (28). Therefore, it is important to develop an effective method to mitigate LPS-induced colonic inflammation and the impairment of gut integrity. The objective of this study was to investigate whether β-carotene could be a bioactive compound in modulating LPS-induced inflammation and tight junction protein changes in colonic epithelial cells.

## Materials and Methods

### Chemicals and Reagents

All-trans retinoic acid (Catalog #R2625) and β-carotene (Catalog #C9750) were purchased from Millipore Sigma (Burlington, MA, USA). Fetal Bovine Serum (FBS), Penicillin-Streptomycin (Pen-Strep, 100X), McCoy's 5A medium, and LPS from E. coli serotype EH100 were purchased from Thermo Fisher Scientific (Waltham, MA, USA). Interferon-γ (IFN-γ) was purchased from R&D Systems (Minneapolis, MN, USA). PureLink RNA extraction kit was obtained from Thermo Fisher Scientific, and Novo cDNA kit was purchased from BioVision Inc. (Milpitas, CA, USA). PowerUp SYBR Green Master Mix was purchased from Thermo Fisher Scientific. Nuclear extract kit and ProStain Protein Quantification Kit were obtained from Active Motif (Carlsbad, CA, USA). For western blot, the primary antibodies including TLR4 (category number sc-293072), NF-κB p65 (sc-8008), C-reactive protein (CRP, sc-69770), GAPDH (sc-47724) and β-actin (sc-47778) were mouse monoclonal antibodies and were obtained from Santa Cruz Biotechnology (Dallas, TX, USA). The primary antibodies including claudin-1 (13255), occludin (91131), and CD14 (56082) were rabbit monoclonal antibodies, and were purchased from Cell Signaling Technology (Danvers, MA, USA). The anti-mouse secondary HRP-conjugated antibodies were purchased from Santa Cruz Biotechnology, whereas the anti-rabbit HRP-conjugated antibodies were purchased from Cell Signaling Technology. Precision Plus Protein^TM^ Standards were purchased from Bio-Rad Laboratories (Hercules, CA, USA). IL-6 (RAB0306) and IL-1β (RAB0273) ELISA kits were purchased from Millipore Sigma. TNF-α ELISA kit (DTA00D) was purchased from R&D Systems. For immunocytochemistry (ICC), claudin-1 (D-4) was obtained from Santa Cruz Biotechnology, and the goat anti-mouse secondary antibody Alexa fluor 488 conjugates (A-11029) was purchased from Thermo Fisher Scientific. ImmunoCruz ABC Kit (sc-516216) was purchased from Santa Cruz Biotechnology, and Fluoromount-G Mounting Medium (00-4959-52) was purchased from Thermo Fisher Scientific.

### Cell Culture and Treatment

HT-29 cells (human colorectal adenocarcinoma cell line) were purchased from the American Type Culture Collection (ATCC, HTB-38) (Manassas, VA, USA). HT-29 cells were used between passages 5-25 in this study. These cells were cultured in McCoy's 5A medium containing 10% FBS and 1X Pen-Strep, and were housed in a humidified atmosphere of 95% air and 5% CO_2_ at 37°C. Cells were propagated according to the protocol published previously (29). Briefly, for individual assays, cells were seeded at a density of 2.5 × 10^5^ per well in 2 mL media overnight, followed by IFN-γ pre-treatment at 50 ng/mL for 12 h, and stimulated with 1 μg/mL LPS with or without β-carotene or ATRA treatments at different dosages (1, 10, 100 nM, 1, and 10 μM).

Colonic epithelial cells and supernatants were harvested after 15 h and stored at −20°C. The doses and time points of IFN-γ and LPS treatment were selected based on the previous report (30) and the results of the preliminary study (Supplementary Figure 1).

### RNA Isolation

The extraction of mRNA was performed by employing the PureLink RNA Mini Kit according to the manufacturer's instructions and was previously reported (29). In brief, lysis buffer with 1% 2-mercaptoethanol was used to lyse the cells, and the entire content was transferred to the RNase-free microcentrifuge tubes and washed with 70% ethanol. After centrifugation at 2,000 × *g* for 2 min, the supernatant was transferred to the cartridges for further filtration. Then, the cartridges were washed with two different wash buffers at separate times, and 50 μL nuclease-free water was added to the center of the cartridges to extract the total RNA. SpectraMax QuickDrop Micro-Volume Spectrophotometer (Molecular Devices, LLC - San Jose, CA, USA) was utilized to examine RNA quality and quantity.

### cDNA Synthesis and Quantitative PCR (qPCR)

cDNA was synthesized from 500 ng RNA samples using the Novo cDNA kit and Biometra TAdvanced 96G Thermal Cycler System (Analytik Jena – Jena, Germany) as reported previously (29). The program conditions were 25°C for 10 min, 42°C for 50 min, and 85°C for 5 min. The detection of mRNA of each sample was carried out by mixing 10 μL 2X PowerUp SYBR Green Master Mix, 2 μL of 10 μM primer mix that includes forward and reverse primers, 3 μL DNase-free water, and 5 μL standardized cDNA. Cycling conditions were 50°C for 2 min and 95°C for 10 min; followed by 40 cycles at 95°C for 15 s, 60°C for 15 s; then 95°C for 15 s, 60°C for 1 min and 95°C for 15 s. Primers were designed using the Primer-BLAST tool at NCBI. Primer sequences were list in Supplementary Table 1.

### Whole Cell/Nuclear Protein Extraction and Western Blot

Whole cell lysates were extracted by using radioimmunoprecipitation assay (RIPA) buffer containing 1% protease inhibitors. Protein concentration was determined by the Bicinchoninic Acid (BCA) Method according to manufacturer instructions.

Nuclear protein was extracted by utilizing the Nuclear Extract Kit as instructed by the manufacturer. Briefly, cells were scraped with ice-cold PBS with phosphatase inhibitors and centrifuged at 200 × *g* at 4°C. Subsequently, Hypotonic Buffer and Detergent were added to the resuspended cells, followed by centrifugation for 30 s. at 14,000 × *g* at 4°C. After removal of the supernatant (cytoplasmic fraction), the nuclear pellets were suspended in Complete Lysis Buffer and incubated for 30 min. on ice. Then, the mixture was centrifuged for 10 min at 14,000 × *g*, and the supernatant (nuclear fraction) was collected and stored at −80°C. Nuclear protein concentration was quantified by using the ProStain Protein Quantification Kit according to the manufacturer's instructions. The fluorescence was measured at 488 nm (excitation) and 635 nm (emission).

To examine specific protein expression, both whole cell lysates and nuclear protein were standardized with molecular biology grade water and mixed with a NuPAGE LDS Sample Buffer (4x) and NuPAGE Reducing Agent (10x). The mixture was then incubated at 70°C for 10 min. To detect the tight junction proteins, 40 μL protein was loaded to the gel, whereas 15–20 μL protein was loaded to detect other proteins by using the electrophoresis. Whole protein extracts of A431 cells (an epidermoid carcinoma cell line) provided by Cell Signaling Technology (Danvers, MA, USA) was used as positive control for the detection of tight junction proteins. Then, proteins were transferred to a nitrocellulose membrane by using the iBlot2 dry transfer system. The membranes were non-specifically blocked with 5% Bovine Serum Albumin (BSA) for 1 h, followed by primary antibody incubation at 4°C overnight. Secondary antibodies were applied to the membranes that were blocked with non-HRP conjugated primary antibodies. Chemiluminescent reagents were added to the membranes to detect the signals. Protein expressions were detected by using a UVP ChemStudio imaging system (Analytik Jena). The target protein bands were at the expected positions within the blots and non-specific bands were not observed; therefore, we are confident that the antibodies' target was displayed as specified by the manufacturer.

### Enzyme-Linked Immunosorbent Assay (ELISA)

Supernatants were collected from cultured cells and stored in −20°C. Quantitative measurement of IL-6, IL-1β, and TNF-α in both supernatants and whole cell lysates (WCL) was conducted according to the manufacturer's instructions of the commercialized human ELISA kits, which were kit-specific to our cytokines of interest. The optical density of each sample was assessed by using the Synergy H1 microplate reader (BioTek, Winooski, VT, USA).

### Immunocytochemistry (ICC)

The intensity and localization of claudin-1 were assessed by immunofluorescent antibody labeling. Briefly, cells on the coverslips were fixed with 4% paraformaldehyde for 10 min at room temperature, followed by washing using ice-cold PBS. Then, the cells were non-specifically blocked with 1% BSA and 22.53 mg/mL glycine in PBST (PBS + 0.1% TWEEN 20) for 30 min. After rinsing with PBS for 3 times, the cells were incubated in primary antibody (1:10 diluted in 1% BSA) overnight, followed by incubation in the secondary antibody Alexa fluor 488 conjugate (1:10 diluted in 1% BSA) for 1 h at room temperature in the dark. The cells were then incubated in a reagent mixture from ImmunoCruz ABC Kit for 15 min to amplify the signals. Fluoromount-G Mounting Medium with 4′,6-diamidino-2-phenylindole (DAPI) was used to mount the coverslips to the slides as well as provide a blue nuclear counter stain. Immunolocalizations of claudin-1 was visualized using a ZEISS LSM710 confocal fluorescence microscope (Carl Zeiss AG, Oberkochen, Germany) equipped with a ZEISS camera.

### Statistical Analysis

Normality of distribution of the total samples was examined by utilizing the D'Agostino-Pearson omnibus normality test. If *p* > 0.05, the data were considered as normally distributed. Equality of variance of data within each group was determined by using an F test. If *p* > 0.05, the data was considered as equal variances. Student's *t-test* was performed to compare the mean of the control group to the mean of the treatment group. A *p* < 0.05 was an indication of statistical significance. Each experiment was carried out in quadruplicates to ensure reproducibility.

## Results

### β-Carotene Inhibited LPS-Induced Inflammation

**Figure 1:**
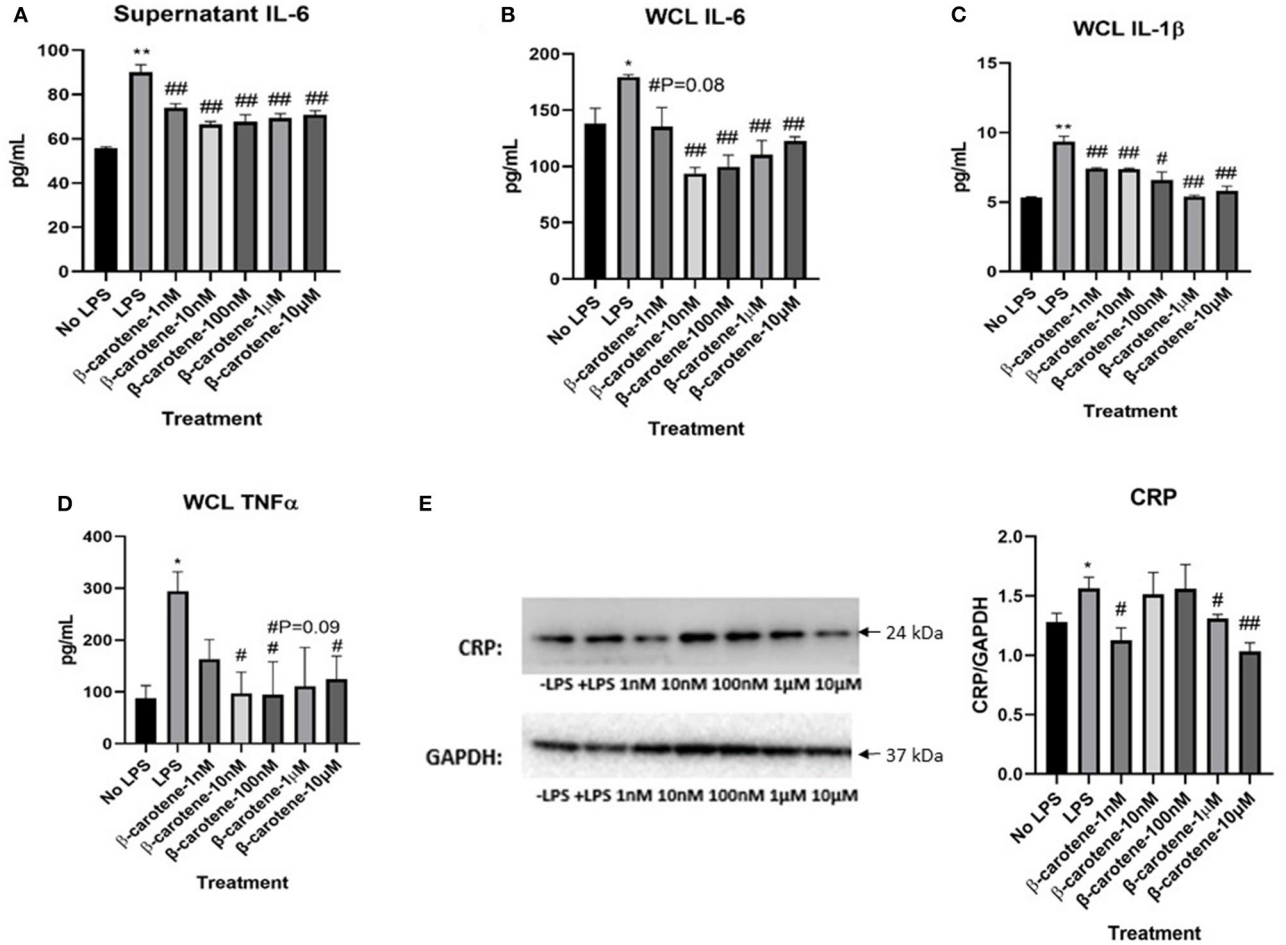
Inhibitory effect of β-carotene on LPS-induced inflammatory markers. **(A–D)** Graphical representations of changes in pro-inflammatory cytokines detected via ELISA: **(A)** Cytokine levels of IL-6 in HT-29 cell culture supernatants; **(B)** IL-6 levels in HT-29 whole cell lysates (WCL); **(C)** IL-1β levels in WCL; **(D)** TNF-α levels in WCL. **(E)** Western blot of CRP expression in WCL. Five treatment doses of β-carotene (1, 10, 100 nM, 1, 10 μM). Four replicates were used for statistical analysis in ELISA and western blot. Values are means ± SEMs. *Significance at *p* < 0.05 for comparison with the “No LPS” group, **significance at *p* < 0.01 for comparison with the “No LPS” group. ^#^significance at *p* < 0.05 for comparison with the “LPS” group, ^##^significance at *p* < 0.01 for comparison with the “LPS” group.

The levels of pro-inflammatory cytokines (IL-6, IL-1β, and TNF-α) were determined using ELISA procedures in both the cell culture supernatants and whole cell lysates (WCL) in HT-29 cells treated with β-carotene. LPS stimulation significantly increased the levels of IL-6 in both supernatant (Figure 1A) and WCL (Figure 1B) of these cells compared to the cells not treated with LPS (“No LPS”) as expected. β-Carotene treatment subsequently decreased IL-6 levels in all treatment groups (1 nM, 10 nM, 100 nM, 1 μM, and 10 μM) in the supernatant samples and nearly all treatments in the WCL compared to the LPS stimulated control; even at the 1 nM β-carotene treatment group, the level of IL-6 decreased, although the *p-*value was not significant.

Furthermore, LPS stimulation significantly increased IL-1β levels compared to “No LPS” as expected (Figure 1C). β-Carotene treated WCL showed a significant decrease in IL-1β cytokine levels at all concentrations compared to the LPS control. Supernatant levels of IL-1β were too low to be detected via ELISA. Finally, LPS stimulation significantly increased TNF-α levels compared to “No LPS” (Figure 1D), as seen with the other cytokines. Although supernatant levels of TNF-α were too low to be quantified via ELISA, β-carotene treated WCL showed a significant decrease in TNF-α cytokine levels at most concentrations in comparison to the LPS control. Additionally, β-carotene treatment showed a non-significant decreasing trend at 1 μM. The ELISA results indicated that β-carotene had an inhibitory effect on the LPS-stimulated release of these pro-inflammatory cytokines. Also, CRP in HT-29 WCL was significantly increased upon LPS stimulation relative to the untreated control, and β-carotene decreased CRP at 1 and 10 μM (Figure 1E). Interestingly, 1 nM β-carotene was able to significantly decrease CRP expression, but 10 and 100 nM did not reach statistical significance; in this case, this phenomenon may not be dose-dependent.

### β-Carotene Improved Tight Junction Proteins

**Figure 2:**
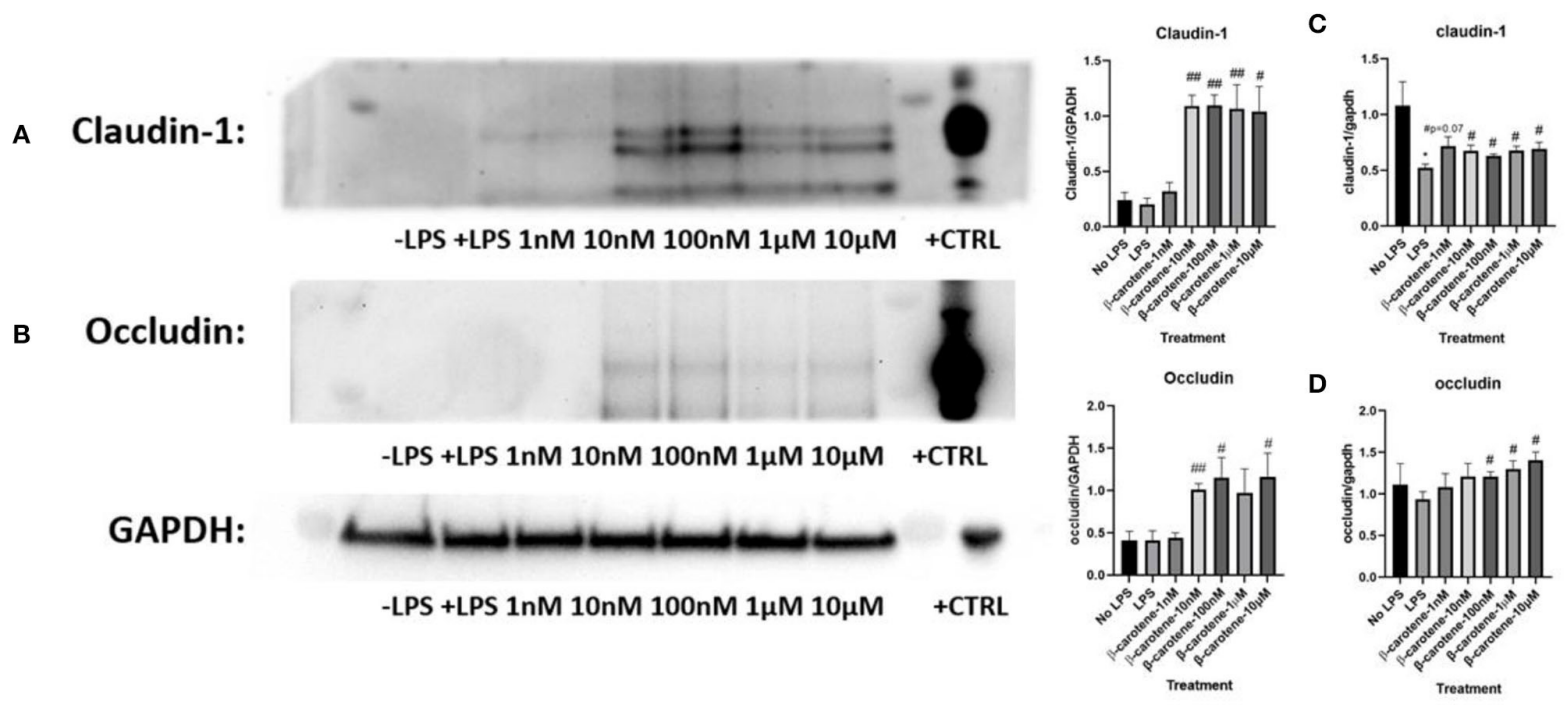
β-Carotene treatments induce tight junction proteins. **(A,B)** Western blot membranes with tight junction protein expression of **(A)** claudin-1 and **(B)** occludin. Positive control (+CTRL), obtained from A431 cells. **(C,D)** Fold changes in mRNA levels of **(C)** claudin-1 and **(D)** occludin generated from qPCR. Five treatment doses of β-carotene (1, 10, 100 nM, 1, 10 μM). Four replicates used for statistical analysis of western blot and qPCR. Values are means ± SEMs. *significance at *p* < 0.05 for comparison with the “No LPS” group. ^#^significance at *p* < 0.05 for comparison with the “LPS” group, ^##^significance at *p* < 0.01 for comparison with the “LPS” group.

We explored the efficacy of β-carotene treatment in modulating tight junction proteins. Regarding claudin-1, the expected decrease of expression upon LPS stimulation was not significantly shown (Figure 2A). However, the expression of claudin-1 was drastically increased with the medium to high β-carotene doses of 10, 100 nM, 1 and 10 μM. These substantial changes due to β-carotene treatment at these concentrations were also significant compared to the baseline levels (“No LPS”) of claudin-1 expression. There was a similar trend with occludin expression (Figure 2B). The positive control, obtained from the epidermoid carcinoma cell line (A431), demonstrated a strong expression of both claudin-1 and occludin in comparison to HT-29 cells indicating that HT-29 cells might have the low endogenous expression of tight junction proteins. We also assessed the mRNA levels of *claudin-1* and *occludin* in the same *in vitro* model. The LPS only treatment group led to a significant decrease in *claudin-1* levels, whereas the β-carotene treatment provided an overall significant increase in *claudin-1* at most concentrations, with the exception for the non-significant increase at 1 nM (Figure 2C). Similar results were observed regarding levels of *occludin* as LPS treated samples showed a non-significant decrease and β-carotene treated samples resulted in increased levels, with significant increases at higher doses of 100 nM, 1, and 10 μM (Figure 2D).

**Figure 3:**
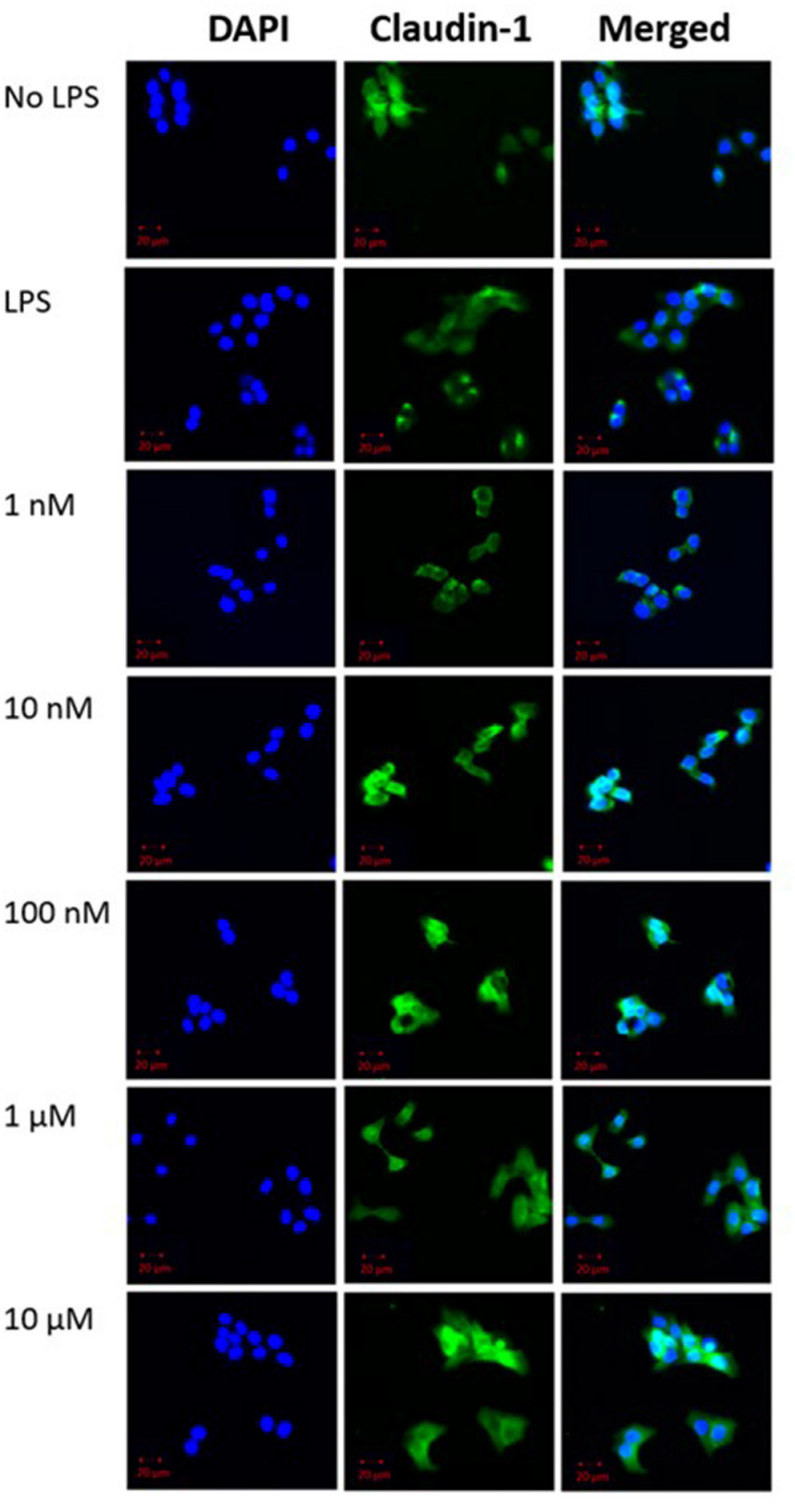
β-Carotene promotes claudin-1 protein expression. Confocal microscope images of claudin-1 protein expression. (DAPI panel) HT-29 cell nuclei indicated via DAPI staining. (Claudin-1 panel) Protein expression of claudin-1 expression portrayed by fluorescent staining via Alexa Fluor 488. Panels are merged in the final panel for visualization of localization. Five treatment doses of β-carotene (1, 10, 100 nM, 1, 10 μM). Scale bar of 20 μm was included for size comparison.

Qualitative analysis of confocal fluorescence microscopy images was obtained to verify the effects of β-carotene on tight junction protein expression changes in HT-29 cells (Figure 3). LPS decreased the expression of claudin-1 as expected, and β-carotene increased the claudin-1 expression across all concentration groups compared to LPS treatment alone. Furthermore, the intensity at the higher end of our treatment range at concentrations of 1 and 10 μM appeared to be stronger than that of the “No LPS” group. These results were correlated with the western blotting results and indicated that β-carotene could upregulate claudin-1 tight junction levels.

### β-Carotene Metabolism—Its Cellular Uptake and Conversion

β-Carotene is a provitamin-A carotenoid, so its metabolite ATRA, the biologically active form of vitamin A, is of particular interest. We first determined its uptake into the colonic epithelial cells via checking SR-B1 levels under our treatment conditions, and there were no observed significant differences of *srb1* mRNA levels between “No LPS” and LPS stimulated groups (Supplementary Figure 2A). Likewise, there were no significant changes of *srb1* expression across the β-carotene treated samples compared to the LPS stimulated group. Therefore, SR-B1 was not significantly affected by the presence of LPS or β-carotene. Furthermore, we examined the expression of BCO1 in our model, and upon LPS stimulation, there was a significant increase in *bco1* mRNA (Supplementary Figure 2B). However, β-carotene did not significantly affect *bco1* mRNA expression except for a downregulation at the 10 μM treatment group, which could indicate that β-carotene may not significantly hinder the BCO1 related cleavage under LPS conditions.

### ATRA Did Not Alter LPS-Induced Inflammation

**Figure 4:**
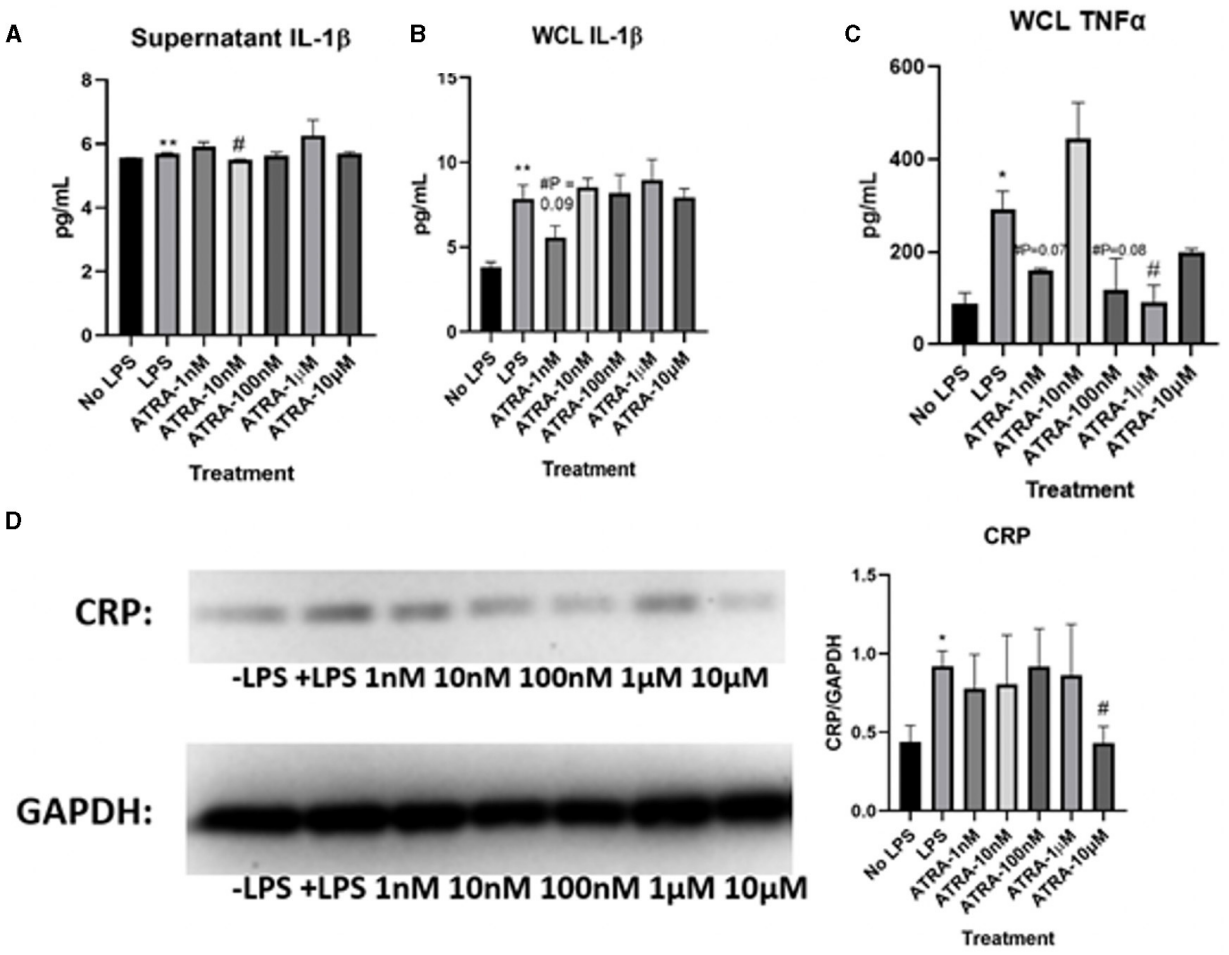
ATRA minimally affects LPS-induced inflammatory markers. **(A–C)** Graphical representations of fold changes in pro-inflammatory cytokines detected via ELISA: **(A)** Cytokine levels of IL-1β in HT-29 cell culture supernatants; **(B)** IL-1β levels in HT-29 whole cell lysates (WCL); **(C)** TNF-α levels in WCL. **(D)** Western blot of CRP expression in WCL. Five treatment doses of ATRA (1, 10, 100 nM, 1, 10 μM). Four replicates were used for statistical analysis in ELISA and western blot. Values are means ± SEMs. *significance at *p* < 0.05 for comparison with the ‘No LPS’ group, **significance at *p* < 0.01 for comparison with the “No LPS” group. ^#^significance at *p* < 0.05 for comparison with the “LPS” group.

Determining how ATRA modulates tight junction proteins and LPS-induced inflammation was essential to understanding whether the anti-inflammatory nature of β-carotene was due to ATRA or the parent compound, β-carotene. As seen previously, ELISA tests showed that LPS stimulation significantly increased the levels of IL-1β in both supernatant (Figure 4A) and WCL (Figure 4B) samples of these cells compared to that of the cells not treated with LPS. However, no sufficient data was shown in this study to justify that ATRA altered LPS-induced inflammation. In this case, ATRA did not significantly decrease IL-1β supernatant levels, except for at 10 nM (Figure 4A).

We also examined how TNF-α proteins were changed in WCL through ATRA treatments. LPS stimulation significantly increased TNF-α levels compared to “No LPS” (Figure 4C). Yet, ATRA had no observed effects on TNF-α WCL levels, except at 1 μM. Supernatant levels of TNF-α were not detectable via ELISA. Based on these results, ATRA did not inhibit LPS-induced production of cytokines to the extent observed in β-carotene treated HT-29 cells. Additionally, LPS treatment increased CRP expression levels (Figure 4D), yet treatments of ATRA led to observed no changes, except for the significant decrease at 10 μM. Based on these collective results, ATRA appeared to have minimal effect on LPS-induced inflammation within HT-29 cells.

### ATRA Did Not Alter Tight Junction Proteins

**Figure 5:**
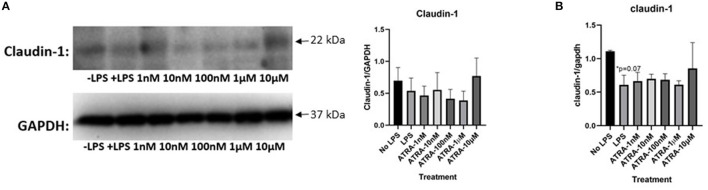
ATRA is not as effective as in causing similar tight junction expression trends as β-carotene. **(A,B)** ATRA treatments on tight junction proteins or mRNA. **(A)** Claudin-1 WCL protein in western blot **(B)** and claudin-1 mRNA expressions in qPCR. Five treatment doses of ATRA (1, 10, 100 nM, 1, 10 μM). Four replicates were used for statistical analysis in western blot and PCR. Values are means ± SEMs. *Significance at *p* < 0.05 for comparison with the “No LPS” group.

To further define the role of ATRA in LPS induced inflammation in our cell model, more experiments were conducted to assess whether it could modulate the expression of tight junction proteins. Like with the β-carotene treated HT-29 cells, expressions of claudin-1 and occludin were assessed with ATRA administration. There was no apparent increase of claudin-1 expression with the treatment of ATRA upon LPS stimulated cells (Figure 5A). Furthermore, the finding of ATRA's minimal effect on claudin-1 was opposite compared to the observations of the β-carotene treated HT-29 cells, as those indicated an increase in claudin-1 expression upon treatment. Likewise, ATRA notably had no effect on occludin across all treatment conditions and failed to improve expression (Supplementary Figure 3A).

Consistently, our qPCR results showed non-significant trends of increasing claudin-1 mRNA levels LPS stimulation and ATRA treatments (Figure 5B). An increase of c*laudin-1* expression was observed at the 10 μM level of ATRA treatment, but not significant in measure. Results from all other treatment arms indicate that ATRA did not affect mRNA for *claudin-1* expression. Additionally, insufficient data was found regarding *occludin* mRNA levels after ATRA treatment (Supplementary Figure 3B). Overall, ATRA administration did not significantly influence tight junction protein modulation.

### β-Carotene Inhibited the TLR4/NF-κB Pathway

**Figure 6:**
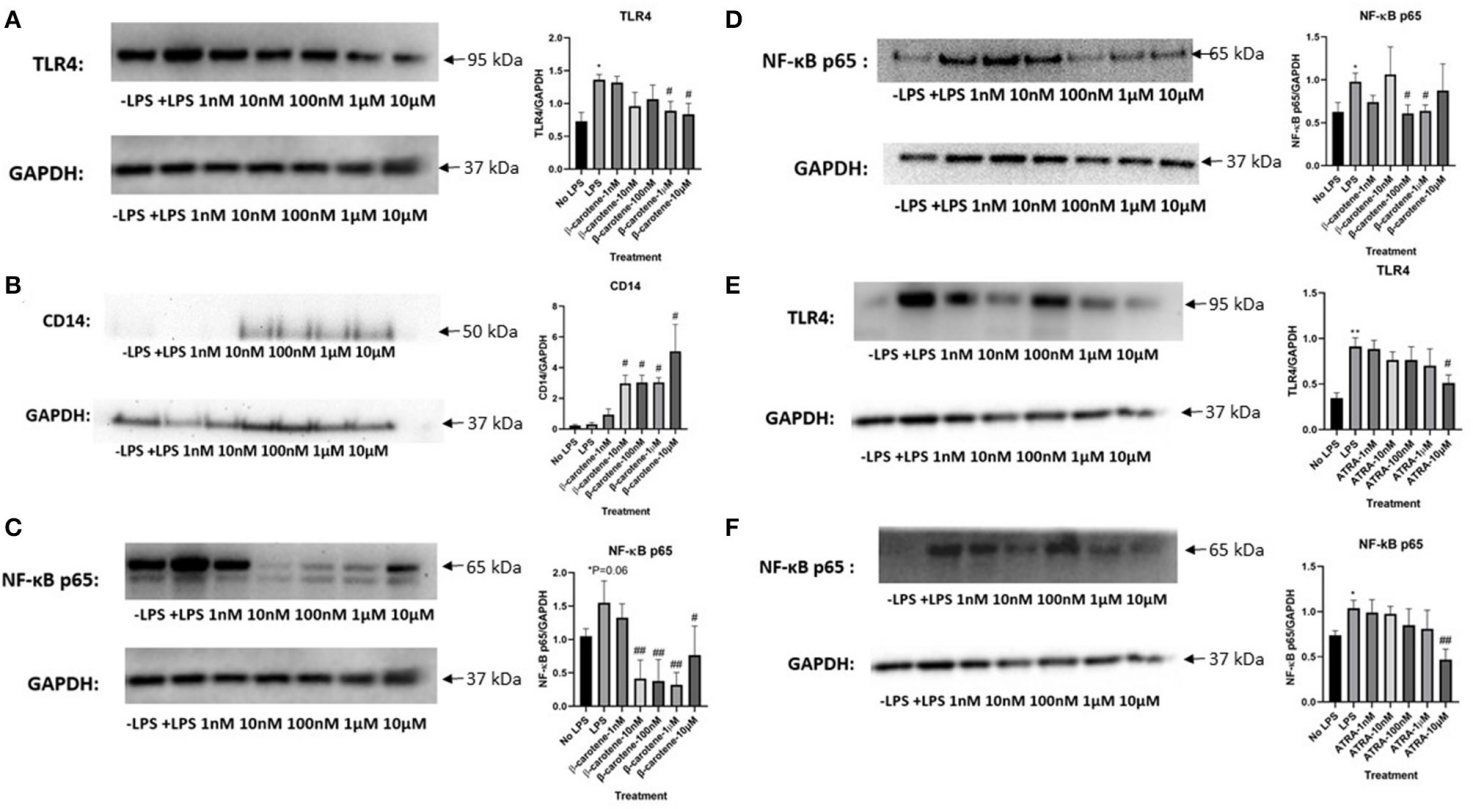
β-Carotene inhibits the TLR4 and NF-κB pathway. **(A–C)** Western blots of β-carotene treated HT-29 WCL-graphical fold changes of **(A)** TLR4 expression, **(B)** CD-14 expression, and **(C)** NF-κB p65. Control images were re-used for illustrative purposes in **(A,C)**. **(D)** Nuclear protein extractions from β-carotene treated samples-western blot and graphical fold changes of **(D)** NF-κB p65 expression. **(E,F)** Western blots of ATRA treated HT-29 WCL proteins-graphical fold changes of **(E)** TLR4 expression and **(F)** NF-κB p65. Four replicates were used for statistical analysis in western blot. Values are means ± SEMs. *Significance at *p* < 0.05 for comparison with the “No LPS” group, **significance at *p* < 0.01 for comparison with the “No LPS” group. ^#^significance at *p* < 0.05 for comparison with the “LPS” group, ^##^significance at *p* < 0.01 for comparison with the “LPS” group.

It is known that elevated levels of LPS promote a pro-inflammatory cascade through activation of toll-like receptor 4 (TLR4) (22). We showed that stimulation of LPS increased TLR4 expression within HT-29 WCL (Figure 6A). β-Carotene was subsequently beneficial toward inhibiting this LPS-induced TLR4 activation at higher treatment dosages of 1 and 10 μM. Previous reports showed beneficial effects of CD14 against inflammatory bowel disease by mitigating inflammation and enhancing intestinal barrier function (31, 32). In the current study, β-carotene concentrations promoted CD14 membrane protein levels at all tested doses, except 1 nM, and such modulation may correlate to regulation of the tight junction claudin-1 and occludin proteins (Figure 6B). As this inflammatory status of HT-29 cells could also be attributed to NF-κB signaling, we investigated the modulation of β-carotene on the NF-κB p65 subunit. Consistent with TLR4 expression, most of the β-carotene treatments led to significant decreases in the protein expression of the NF-κB p65 subunit at concentrations of 10, 100 nM, 1, and 10 μM (Figure 6C). Additionally, we investigated the nuclear translocation of NF-κB p65 within nuclear extracts of HT-29 cells. As expected, LPS treatment significantly increased expression of NF-κB p65 in these nuclear extracts, and β-carotene treatments showed significant decreases seen in treatment arms 100 nM and 1 μM (Figure 6D).

We also tested whether ATRA treatments exerted similar effects in the TLR4/NF-κB pathway. ATRA showed a slight decreasing dose-dependent trend in TLR4 expression in treated samples, albeit non-significant except at 10 μM (Figure 6E). ATRA similarly affected in NF-κB p65 expression (Figure 6F). In conclusion, β-carotene decreased LPS-induced TLR4 activation at higher treatment dosages of 1 and 10 μM, whereas ATRA did not. Likewise, most β-carotene treatments led to a significant decrease in the protein expression of the NF-κB p65 subunit on both WCL and nuclear extracts, but ATRA had no such effect.

## Discussion

In the current study, β-carotene treatment in HT-29 cells reduced LPS-induced inflammation and enhanced tight junction proteins in colonic epithelial cells. In accordance, one *in vivo* study reported that in weaning piglets, β-carotene supplementation significantly decreased serum IL-1β, IL-6, and TNF-α concentrations, compared to the cell without LPS treatment (33). Using the same animal model, the researchers found that β-carotene supplementation resulted in an attenuated jejunal permeability and improved claudin-3, occludin, and zonula occludens protein-1 in the jejunum of piglets that have an increased risk of intestinal stress due to weaning (34). In an ulcerative colitis (UC)-associated mouse model, β-carotene supplementation at 20 mg/kg body weight (BW)/day for 28 days significantly reduced colonic IL-6 and TNF-α levels (35), which was in line with our findings. Another study reported that in β-lactoglobulin-sensitized mice, β-carotene at 5 and 10 mg/kg body weight significantly decreased intestinal inflammation and enhanced intestinal barrier function (36). Yu et al. reported that in rats, β-carotene supplementation suppressed LPS-induced intestinal inflammation via modulating autophagy and regulating the JAK2/STAT3 and JNK/p38 MAPK signaling pathways (37). A recent study depicted the beneficial effects of β-carotene in a DSS-induced UC model by showing decreased colonic levels of pro-inflammatory cytokines, lowered expression levels of phospho-p65, phospho-p38 and reduced activation of Erk and JNK in the rats fed with β-carotene (38). Nevertheless, these were *in vivo* studies that mostly focused on small intestine, whereas we concentrated more on answering fundamental questions about the effects and potential mechanism of β-carotene treatment in modulating LPS-induced inflammation and changes in tight junction protein expression in colon, by particularly using the human epithelial cells.

The efficacy of vitamin A on intestinal barrier function was inconsistently reported. Li et al. showed that in Caco-2 cells, ATRA treatment at 5 μM substantially enhanced transepithelial electric resistance (TEER) and ZO-2 (39). On the contrary, Baltes et al. reported that ATRA treatment at 10 nM-1 μM in Caco-2 cells significantly reduced the TEER values and increased claudin-2, a tight junction protein that decreases barrier function by forming ion pores (40). In the current study, ATRA treatment at 1 nM-10 μM did not show consistent effects in altering colonic inflammatory biomarkers or tight junction protein expressions. The discrepancy between our study and earlier reports might be due to the differentiation status of the cells. It is worth mentioning that plasma ATRA levels in human beings are ~0.57–6.6 ng/mL (41, 42), at least 150-fold less than plasma retinol concentration (41). Such value was substantially lower than the ATRA dosage given to the cells in the previous reports (39, 40). Therefore, future studies may be needed to achieve physiological relevance by supplementing the cells with an even lower dosage of ATRA. We failed to observe a difference of claudin-1 and occludin protein expressions in LPS-treated cells compared to the cells without LPS treatment. Multiple publications have portrayed a loss of tight junction proteins in LPS-treated cells (43–45), but none of these studies investigated the effect of LPS on tight junction protein expressions in intestinal epithelial cells. It has been reported that in mice, the injection of 2 mg/kg LPS induced resulted in gut epithelial barrier dysfunction, which was accompanied by decreased ZO-1 and occludin in the colonic mucosa (46). However, the colonic protein level of claudin-1 was comparable between the control and LPS-treatment groups (46), which was in line with our finding that LPS did not alter claudin-1 protein expression. In Caco-2 cells, LPS treatment induced an increase in tight junction permeability by up-regulating TLR4 (47). In the current study, we found that β-carotene significantly decreased TLR4 protein levels, which was associated with an enhancement of claudin-1 and occludin protein concentrations. One previous study reported that LPS increased cell permeability via an intracellular mechanism involving TLR-4-dependent regulation of CD14 (32). CD14 is a membrane protein that is constitutively expressed on the surfaces of epithelial cells (31) and showed beneficial effects against inflammatory bowel disease by mitigating inflammation and enhancing intestinal barrier function (31, 48). We found increased CD14 protein expression and reduced TLR4 protein expression in the cells treated with β-carotene, indicating the possibility that β-carotene might enhance claudin-1 and occludin protein levels through regulating CD14. Nevertheless, we only showed the mechanistic investigation highlighting the correlation between the TLR4-CD14 pathway and tight junction expressions, not causality. We cannot rule out the possibility that β-carotene alleviated colonic inflammation and improved tight junction proteins through other molecular pathways.

Considering that HT-29 cells are hyporesponsive to LPS (49), we primed the cells with IFN-γ at 50 ng/mL for 12 h before LPS treatment since priming HT-29 cells with IFN-γ can enhance their responsiveness to LPS (49). We selected to treat the cells with LPS at a dosage of at 1 μg/mL for 15 h based on the results of our preliminary study (Supplementary Figure 1). However, it is worth mentioning that in human plasma, LPS concentration ranges from undetectable levels up to 0.2 ng/mL (50–53), even in patients with systemic endotoxemia (50), but with LPS at this range, we were not able to observe increased inflammatory cytokine release or enhanced TLR4 protein expression in HT-29 cells.

Our results showed a reduction of IL-1β and IL-6 in the supernatant with β-carotene treatment at any dosage, and a decrease of TNF-α in the cell WCL with β-carotene treatment at 10, 100 nM, 1, and 10 μM. However, it remains unknown that whether β-carotene decreased the levels of pro-inflammatory cytokines at transcriptional levels, since we failed to detect the mRNA concentrations of these cytokines by using PCR due to their relatively low quantification in the cells.

Regarding the doses that we selected in this study, previous human studies have shown that plasma β-carotene concentration ranges from ~125–425 nM (54, 55). β-Carotene concentration can be between 680 and 2,255 nM in circulation depending on whether it is consumed as a pure compound or within the foods (54, 56). We recently reported that engineered *S. boulardii* synthesized high doses of β-carotene in the colon of mice, and the intake of such engineered probiotics may result in an even higher β-carotene concentration in the gut (57). Altogether, the dosage of β-carotene used in this study is within physiological relevance and can be achieved by humans.

There are several limitations and shortcomings in the current study. First, we could not conduct a TEER assay to explore the LPS-induced changes in cell permeability. A differentiated HT-29 cell line is required to ensure that the cells form a monolayer to conduct TEER assay (58). However, cellular differentiation in HT-29 cells may attenuate their response to LPS by down-regulating TLR4 (59). Furthermore, the differentiated HT-29 cells may express brush-border-associated hydrolases and present brush-border microvilli, which resembles small intestinal cells (58). Since our research focuses on LPS-induced colonic dysfunction, we utilized undifferentiated HT-29 cells, which explains the inability to conduct the TEER assay. We plan to use NCM460D, a normal human colon mucosal epithelial cell line (60), to investigate further the efficacy of β-carotene in inhibiting LPS-induced colonic inflammation and colonic barrier function. Finally, yet importantly, β-carotene can be cleaved eccentrically to form a series of products such as β-apo-14′-carotenoic acid and β-apo-13-carotenone (61). Since earlier reports showed that the metabolites of carotenoids from eccentric cleavage might present various beneficial effects (62–64), we cannot rule out the possibility that compared with a single β-carotene compound, the eccentric cleavage products from β-carotene may play a more prominent role in anti-inflammation and modulate tight junction proteins. Finally, we also will conduct experiments to examine whether other major dietary carotenoids would mitigate inflammation in colon.

**Figure 7:**
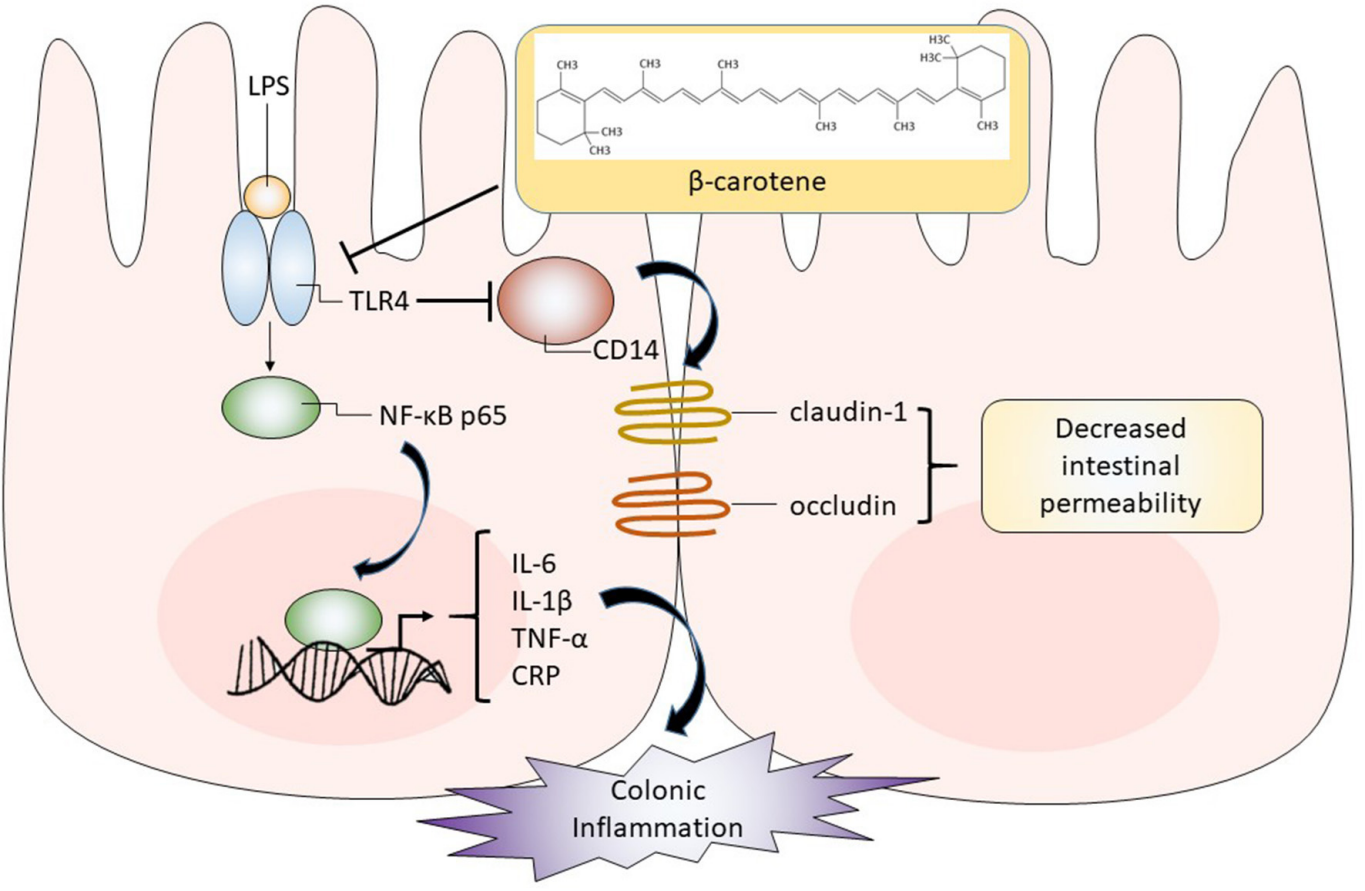
Graphical representation of proposed pathway in which β-carotene impacts colonic epithelial cells. β-Carotene effectively inhibits the LPS-induced inflammatory response within HT-29 colonic epithelial cells by downregulating TLR4, which may block the activation and nuclear translocation of the NF-κB p65 subunit and related downstream signaling. Thus, the release of pro-inflammatory cytokines (IL-6, IL-1β, TNF-α) is compromised. The β-carotene induced downregulation of TLR4 may also alleviate the inhibition of its co-receptor CD14. Enhanced CD14 levels by β-carotene may upregulate tight junction proteins (claudin-1 and occludin), leading to decreased intestinal permeability and enhanced barrier integrity.

Taken together, we provided the first evidence that β-carotene treatment, even at low dosages, can inhibit LPS-induced colonic inflammation and enhanced the expression of tight junction proteins possibly by down-regulating the TLR4 pathway as summarized in Figure 7. Further *in vitro* mechanistic studies and *in vivo* studies are warranted.

## Data Availability Statement

The original contributions generated for the study are included in the article/Supplementary Material, further inquiries can be directed to the corresponding author/s.

## Author Contributions

JC, EB, and AE: conceptualization, formal analysis, investigation, and supervision. JC, EB, BM, and AE: data curation, methodology, resources, software, validation, visualization, and writing original draft. AE: funding acquisition and project administration. All authors have read and agreed to the published version of the manuscript.

## Funding

This work was supported by the USDA National Institute of Food and Agriculture, [Hatch] project [accession number #1021933] and National Science Foundation Grant 1643814.

## Conflict of Interest

The authors declare that the research was conducted in the absence of any commercial or financial relationships that could be construed as a potential conflict of interest.

## Publisher's Note

All claims expressed in this article are solely those of the authors and do not necessarily represent those of their affiliated organizations, or those of the publisher, the editors and the reviewers. Any product that may be evaluated in this article, or claim that may be made by its manufacturer, is not guaranteed or endorsed by the publisher.

## Abbreviations

LPS: lipopolysaccharide
TLR4: toll-like receptor 4
ICC: immunocytochemistry
BCO1: beta-carotene-15, 15′-oxygenase 1
ATRA: all-trans retinoic acid
BCO2: beta-carotene-9, 10′-oxygenase 2
SR-B1: scavenger receptor class B type 1
CRP: C-reactive protein
GI: gastrointestinal
PRR: pathogen recognition receptor
IFN-γ: Interferon-γ
IL-6: Interleukin-6
IL-1β: Interleukin-1β
TNF-α: Tumor Necrosis Factor-α
BCA: Bicinchoninic Acid
DAPI: 4′, 6-diamidino-2-phenylindole
WCL: whole cell lysate.
