# Supplementary material for: The Role of β-Carotene in Colonic Inflammation and Intestinal Barrier Integrity
Source: Front Nutr. 2021 Sep 27;8:723480. doi: 10.3389/fnut.2021.723480 (PMC8502815; doi:10.3389/fnut.2021.723480)
Supplement: Supplementary file 1 [file Data_Sheet_1.docx]

Supplementary Material

**Supplementary Data**

**
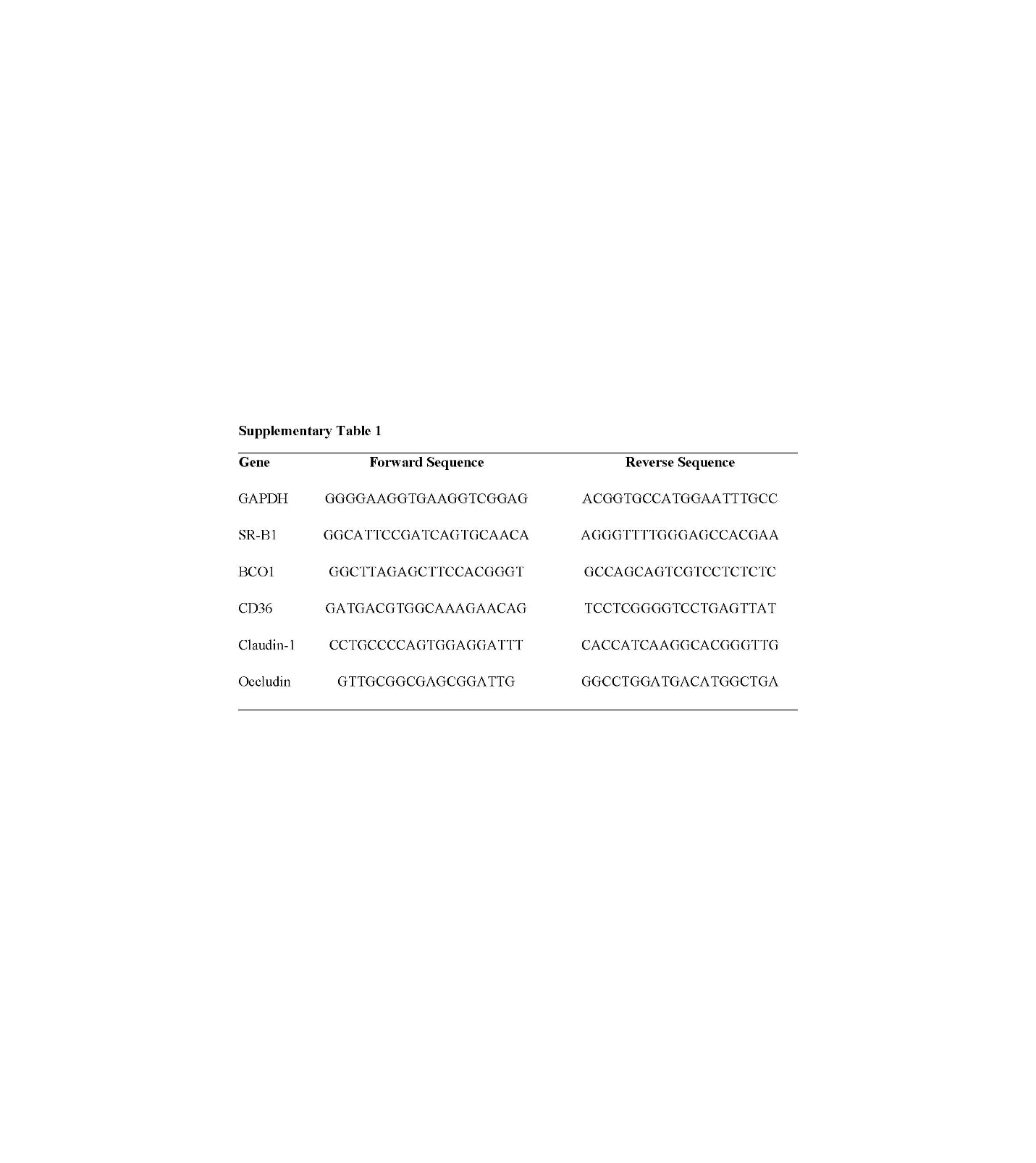
**

***Supplementary Table 1: Primer sequences used in qPCR experiments.*** Forward and reverse sequences generated with the NCBI BLAST tool.

**
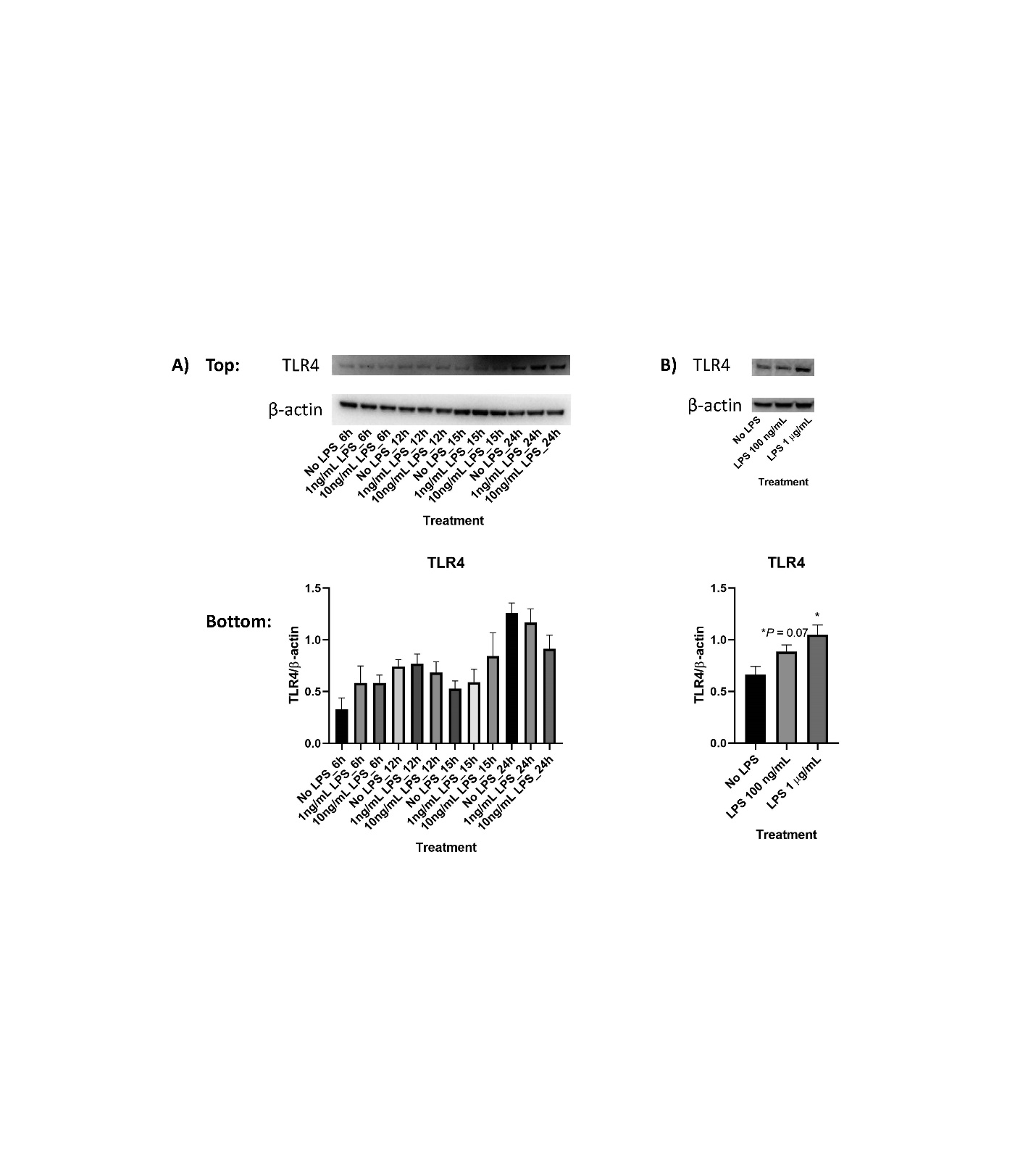
**

***Supplementary Figure 1: Selection of LPS treatment conditions.*** **A)** Time course and dose response for LPS treatment-four time points (6hr, 12hr, 15hr, 24hr) and three dosages (No LPS, 1 ng/mL, 10 ng/mL). **Top-**western blot of TLR4 expression in HT-29 WCL; **Bottom-**fold changes of TLR4. **B)** LPS dose response at 15hr, three dosages (“No LPS”, 100 ng/mL, 1 μg/mL). **Top**- western blot of TLR4 expression; **Bottom-** fold changes of TLR4. Four replicates were used for statistical analysis in western blot. Values are means ± SEMs. *indicates significance at *p* *<* 0.05 for comparison of cells not treated with LPS (No LPS) versus cells treated with LPS.

**
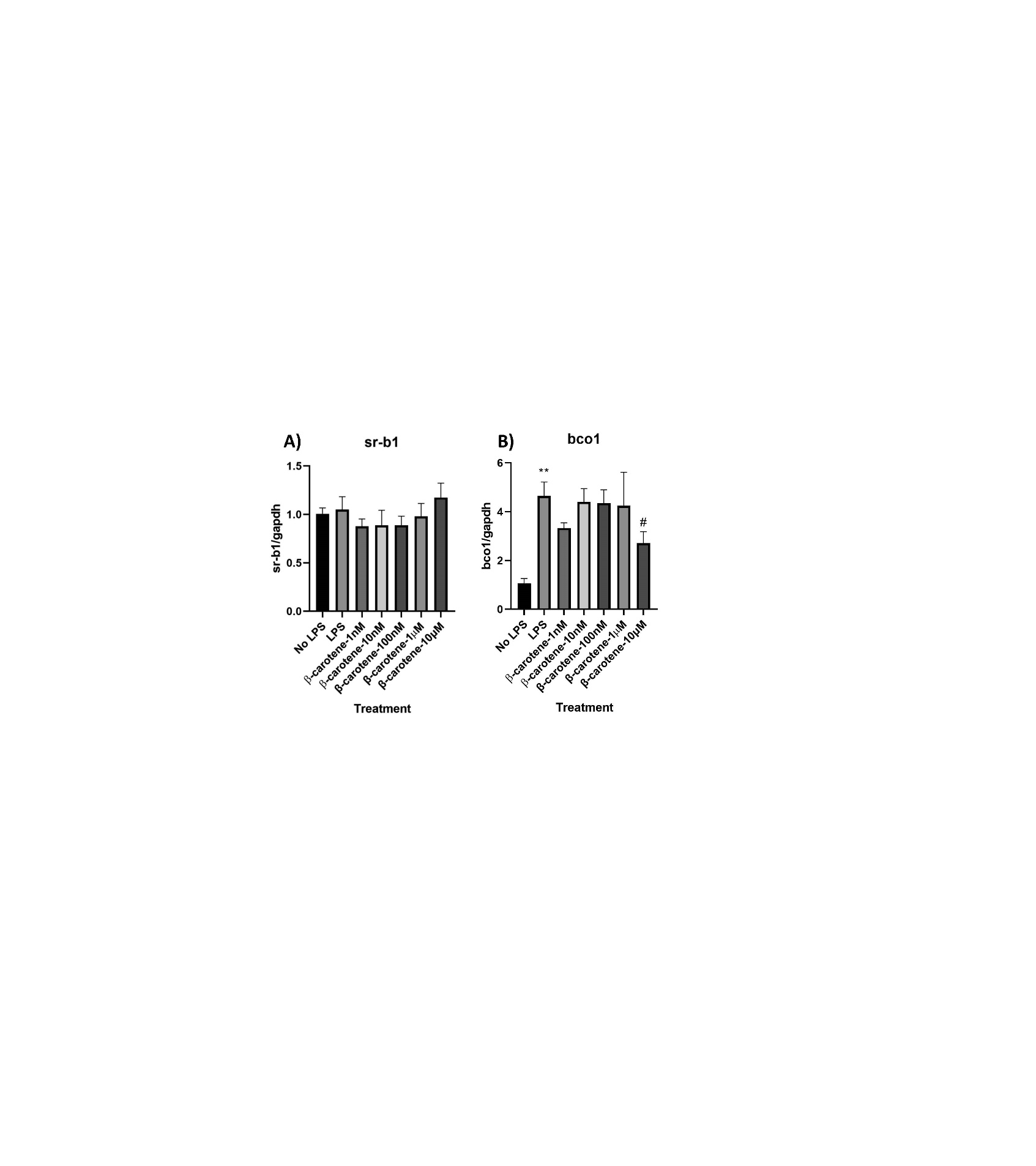
**

***Supplementary Figure 2: β-Carotene metabolism in HT-29 cells.*** Fold changes in mRNA levels of **A)** sr-b1 **B)** bco1 Five treatment doses of β-carotene (1 nM, 10 nM, 100 nM, 1 µM, 10 µM). Four replicates used for statistical analysis of qPCR. Values are means ± SEMs. **indicates significance at *p <* 0.01 for comparison with the “No LPS” group. #indicates significance at *p* *<* 0.05 for comparison with the “LPS” group.

**
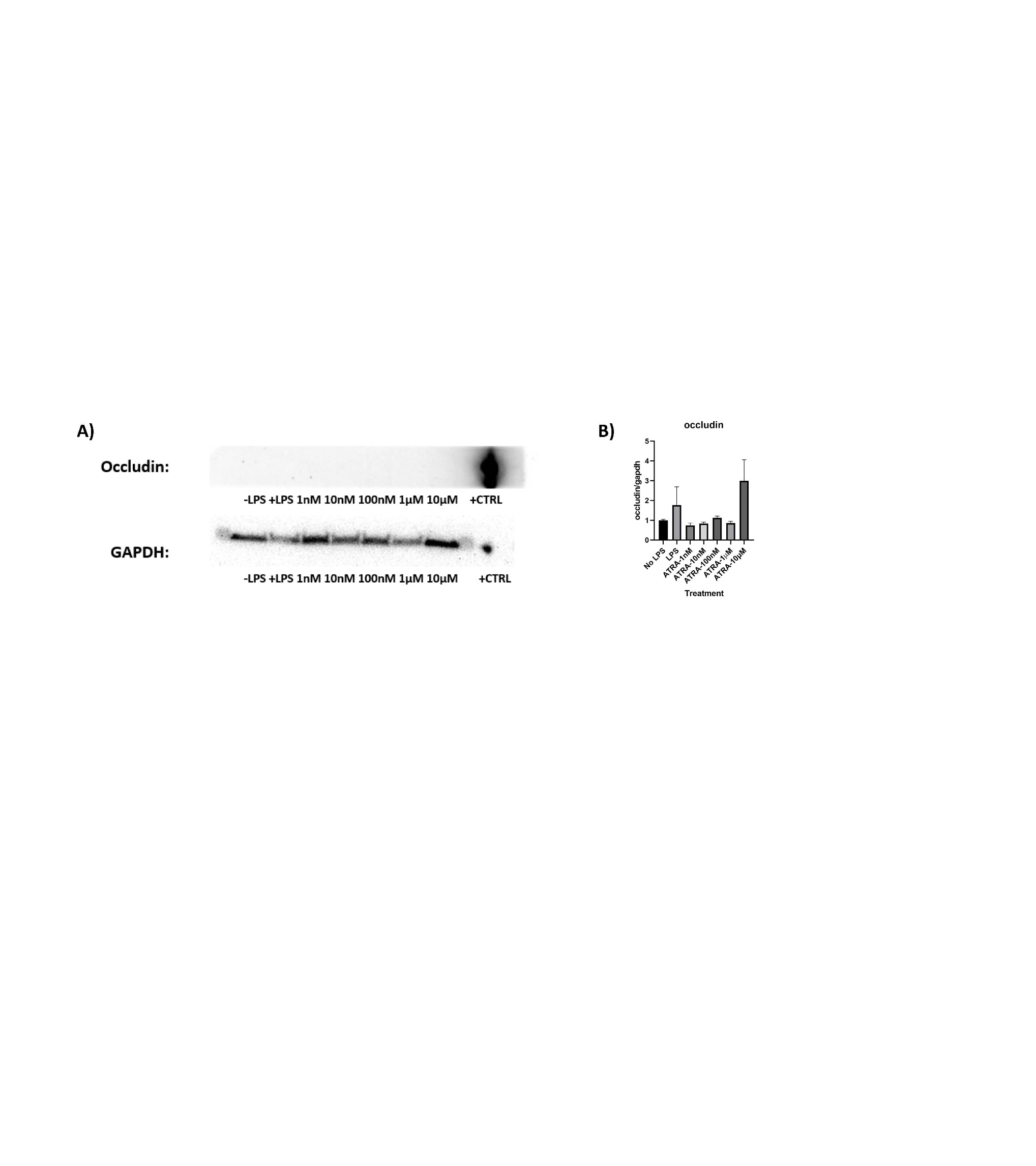
**

***Supplementary Figure 3: The effect of ATRA in occludin protein expression***. **A)** Western blot of occludin protein levels in ATRA treated HT-29 WCL. Positive control (+CTRL), obtained from A431 cells. No statistical analysis done due to lack of occludin expression. **B)** Graphical fold changes of occludin mRNA levels in ATRA treated HT-29 cells obtained via qPCR. Four replicates were used for statistical analysis in western blot and qPCR. Values are means ± SEMs.
